# Sub-national levels and trends in contraceptive prevalence, unmet need, and demand for family planning in Nigeria with survey uncertainty

**DOI:** 10.1186/s12889-019-8043-z

**Published:** 2019-12-30

**Authors:** Laina D. Mercer, Fred Lu, Joshua L. Proctor

**Affiliations:** Institute for Disease Modeling, Bellevue, Washington USA

**Keywords:** Modern contraceptive prevalence rate, Demand satisfied, Bayesian hierarchical model, Small area estimation, parity

## Abstract

**Background:**

Ambitious global goals have been established to provide universal access to affordable modern contraceptive methods. To measure progress toward such goals in populous countries like Nigeria, it’s essential to characterize the current levels and trends of family planning (FP) indicators such as unmet need and modern contraceptive prevalence rates (mCPR). Moreover, the substantial heterogeneity across Nigeria and scale of programmatic implementation requires a sub-national resolution of these FP indicators. The aim of this study is to estimate the levels and trends of FP indicators at a subnational scale in Nigeria utilizing all available data and accounting for survey design and uncertainty.

**Methods:**

We utilized all available cross-sectional survey data from Nigeria including the Demographic and Health Surveys, Multiple Indicator Cluster Surveys, National Nutrition and Health Surveys, and Performance, Monitoring, and Accountability 2020. We developed a hierarchical Bayesian model that incorporates all of the individual level data from each survey instrument, accounts for survey uncertainty, leverages spatio-temporal smoothing, and produces probabilistic estimates with uncertainty intervals.

**Results:**

We estimate that overall rates and trends of mCPR and unmet need have remained low in Nigeria: the average annual rate of change for mCPR by state is 0.5% (0.4%,0.6%) from 2012-2017. Unmet need by age-parity demographic groups varied significantly across Nigeria; parous women express much higher rates of unmet need than nulliparous women.

**Conclusions:**

Understanding the estimates and trends of FP indicators at a subnational resolution in Nigeria is integral to inform programmatic decision-making. We identify age-parity-state subgroups with large rates of unmet need. We also find conflicting trends by survey instrument across a number of states. Our model-based estimates highlight these inconsistencies, attempt to reconcile the direct survey estimates, and provide uncertainty intervals to enable interpretation of model and survey estimates for decision-making.

## Background

International support for improving access to family planning (FP) services has had a significant resurgence in the past decade. Ambitious, world-wide goals have been constructed by coalitions of governmental and non-governmental agencies. One such goal, colloquially referred to as *120 by 20*, aims to increase access to modern contraceptives for 120 million more women by 2020; architects of *120 by 20* included principals from the Bill and Melinda Gates Foundation (BMGF), the United Kingdom Department for International Development, the United States Agency for International Development (USAID), and the United Nations Population Fund [1]. Identified at the inception of this goal, a major barrier to achieving *120 by 20* was the determination of baseline of modern contraceptive prevalence rates (mCPR) in 2012 and ability to track yearly progress [1]. Similarly, the more recently developed sustainable development goal (SDG) 3.7.1 http://sustainabledevelopment.un.org/owg.html of increasing demand satisfied, defined as the ratio of mCPR to total contraceptive prevalence and unmet need, requires reliable estimates of FP indicators. To meet those challenges, substantial investments in new measurement instruments [2] and novel model-based estimate methodologies [3, 4] enabled the establishment of a baseline and a methodology for estimating yearly national progress.

Unfortunately, the progress toward *120 by 20* is currently falling short with only 38.8 million estimated additional users since 2012 [5]. With few exceptions [6], national mCPR rates have not increased nor accelerated as planned [1], especially in priority settings such as Nigeria which have not observed substantial national increases in mCPR despite explicit country goals to increase by more than 1.5*%* per year [7]. To enable within country decision-making around increasing mCPR to meet the current needs of women, a finer-scale approach to evaluating progress and the impact of FP programs is essential. Achieving global goals, whether *120 by 20* or SDG 3.7.1, will require continued international commitment with specific, targeted family planning programs. In this article, we develop a model-based estimation approach to characterize FP indicators at a *sub-national scale*. Here, we focus on Nigeria to demonstrate how our Bayesian hierarchical model integrates all available cross-sectional survey data from a diverse set of measurement instruments and incorporates measurement uncertainty to produce state-level estimates and uncertainty intervals for FP indicators.

Previous analyses and model-based estimates by Alkema et al. [3] have focused on national level estimates of mCPR and unmet need. Their innovative modeling methodology is the foundation of the family planning estimation tool (FPET) [4], which is being used to track progress toward the *120 by 20* goals [5]. FPET has also been utilized to estimate FP indicators for 29 states and union territories in India, where large-sample, cross-sectional surveys have been performed [8]. The model in [3] assumes a parametric form of a logistic growth curve for mCPR increases; the assumption is founded in the theory of social diffusion of ideas for family planning services [9] and has enabled long-term forecasts of FP indicators matching population growth estimates in the next century [10]. However, for sub-national estimates in settings where progress has stalled or even *declined*, the parametric assumption in [3] is not consistent with the data and can strongly influence estimates and trends. Moreover, coupled with a lower density of measurements at a subnational level and higher in-sample uncertainty, the modeling methodology in [3] is lacking in it’s ability to account for measurement uncertainty, especially when also considering demographic subgroups beyond all married women.

In contrast, small area estimation (SAE) methodologies combined with Bayesian hierarchical modeling can effectively combine survey and spatial statistics; this approach has been used to estimate sub-national childhood mortality from multiple household surveys and health and demographic surveillance system sites [11]. Broadly, these Bayesian hierarchical models inherit several important aspects of modern estimation methodologies: uncertainty is quantified for each estimate [12], data is integrated from multiple surveys and designs [11, 13], and spatio-temporal random effects are included in the model [14]. SAE techniques have been previously implemented for FP indicators restricted to data collected from Performance Monitoring and Accountability 2020 (PMA2020) surveys for ten low-income countries [15]. However, this effort did not include data from other household surveys nor was PMA2020 nationally representative for all survey rounds in Nigeria. The aim of our study is substantially broader: we produce state-level estimates of FP indicators while incorporating large-scale household surveys (the Demographic and Health Surveys (DHS), Multiple Indicator Cluster Surveys (MICS), National Nutrition and Health Surveys (NNHS), and PMA2020), include survey specific effects in the model, and generate uncertainty intervals for all estimates across the 36 Nigerian states and the Federal Capital Territory. Moreover, the underlying model can be easily generalized to other FP priority countries and adapted to estimating FP indicators for demographic subgroups such as age and parity.

Significant international resources and investments are being directed to Nigeria to meet the current demand for family planning services. For example, external donors, such as BMGF and USAID, fund non-governmental groups to implement family planning programs and improve supply chain; these donors also work closely with the Nigerian government to rollout new contraceptive technologies such as the new self-injection Depo-Medroxyprogesterone Acetate - subcutaneous (DMPA-SC). Each of these are implemented or rolled-out at sub-national spatial scales. Our modeled estimates and uncertainty intervals provide specific feedback to country programs: new survey data (i.e., the upcoming 2018 DHS) can be contextualized across all surveys avoiding mis-interpretation of uncertain estimates that may naïvely suggest large increases or decreases; estimates and uncertainty intervals offer a basis to interpret the expected effect of investments and programs over time; and analysis of age-parity subgroups helps characterize the demand for services.

## Methods

### Cross-sectional survey data

**Fig. 1:**
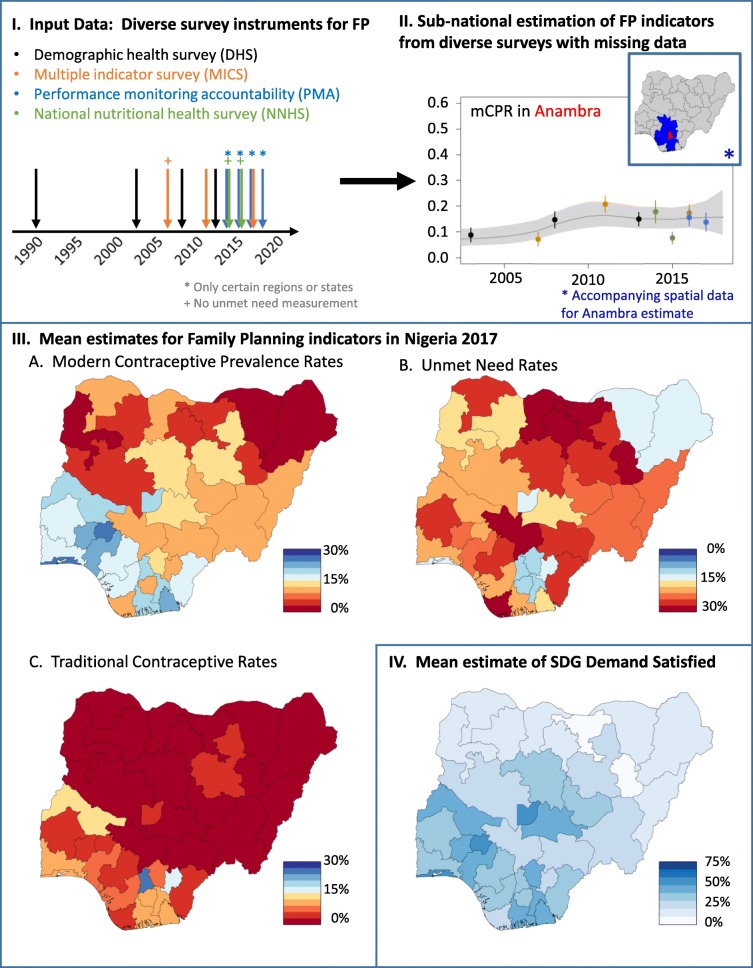
Description of data included (I), example of direct and model-based estimates of mCPR in Anambra state (II), maps of posterior median mCPR, unmet need, and traditional contraceptive prevalence rate for 2017 (III), and posterior median of demand satisfied for 2017 (IV)

Data comes from four DHS, three MICS, and two NNHS, each of which are nationally representative household surveys based on multi-stage cluster survey design. The four PMA2020 rounds which include two to seven states were also integrated into the analysis. We utilize all available DHS, MICS, NNHS, and PMA2020 surveys from Nigeria; see Additional file 1 (Section 1) for specific details about the survey, year, complex design, and survey-to-survey inconsistencies. Figure 1(I) illustrates the year each survey was performed. For each survey, we extract individual level data describing modern or traditional contraceptive usage status, the revised definition of unmet need [16], demographic information such as age and parity, survey design variables, and location information such as state. We also used the associated spatial data publicly available by DHS [17], which includes subnational spatial data such as state shapefiles, enabling both the small area estimation analysis and the illustration of analytic results in the form of subnational maps of Nigeria; the github repository [18] contains the R scripts which perform the analysis and generate the maps of Nigeria contained in this article.

### Analyzing rates of contraceptive usage and unmet need

Traditional and modern contraceptive prevalence rates (mCPR) are measured by computing the percentage of women who report themselves or partners as using at least one contraceptive method. Unmet need for family planning is measured by computing the percentage of women who do not want to become pregnant, either for ending childbearing or delaying the next pregnancy, and are not using a contraceptive method. Here, we use the revised definition of unmet need [16]. FP indicators are computed at the state level with respect to all women as well as four different demographic subgroups: nulliparous women 15−24, parous women 15−24, nulliparous women 25 years or older, and parous women 25 years or older. All surveys included in our analyses provide state level estimates of the mCPR; Fig. 1(II) illustrates the mean estimate and confidence interval of mCPR for each survey in Anambra state for all women.

### Model for sub-national estimation of family planning indicators

Our statistical model assumes direct survey estimates are measurements with associated uncertainty and aims to estimate the underlying rates of FP indicators by state in Nigeria. Our framework is fundamentally a Bayesian hierarchical model allowing for a spatiotemporal smoothing which explicitly integrates multiple surveys, survey designs, and survey uncertainty. In the first stage, the model assumes the pseudo-likelihood defined by the asymptotic distribution of the logit transformed design-based estimates and appropriately transformed design-based variance. At the second stage, the logit transformation of the true FP indicator rates are modeled linearly as a function of independent random effects for short-term fluctuations, a random walk of order 2 to capture national temporal trends, and temporally structured space-time interaction to account for sub-national temporal trends [14]. We also utilize independent and spatially structured random effects to account for effects by sub-national area and provide geographical smoothing [19, 20]. The latter enables data and trends from physically adjacent states to support local estimates. The inset map of Fig. 1(II) illustrates the location of Anambra in Nigeria (red) and the states where data is utilized in the model to support estimates for Anambra (blue). Survey type, survey-time, and survey-space random effects are also considered to account for systematic trends or biases across measurement instruments, time and space. We report medians and 95% credible intervals from the posterior distribution. See Additional file 1 (Section 2) for details on the random effects and hyperparameter priors.

### Fitting the model to data

Models were fit using the R scientific computing language [21]. Direct estimates and design-based variances were calculated using the survey package [22]. Our hierarchical Bayesian space-time model is fit using the Integrated Nested Laplace Approximation (INLA) [23] as implemented in the INLA package in R [24]. INLA provides a fast, efficient, and accurate alternative to Markov Chain Monte Carlo (MCMC) and it has been shown that the approximation is accurate for spatio-temporal smoothing models [25, 26]. Code for all analyses is available at the github repository [18].

### Model selection procedure

We provide a detailed analysis of model structures including evaluating various random effects through principled model selection techniques. Three model selection procedures are considered: the sum of the log conditional predictive ordinate (LCPO) [26], the deviance information criteria (DIC) [27], and the Watanabe-Akaike information criterion (WAIC) [28]. Each model selection criteria aims to evaluate goodness of fit and model complexity; we compute each measure since there has not been consensus on a single criteria [11] and DIC has been shown to under-penalize large models similar to ours [29]. Additional file 1 (Section 3) illustrates each of the proposed models and their relative LCPO, DIC, and WAIC scores.

## Results

### A Bayesian hierarchical model enables state-level estimates of primary FP indicators

Posterior median estimates of the FP indicators for each Nigerian state are shown in Fig. 1(III). Northern states in Nigeria have a much lower mCPR, less than 10%, compared with many of the southern states which exceed 15%. Rates of traditional contraceptive usage are low across most Nigerian states with notable exceptions such as Ebonyi. State level estimates of unmet need are more highly variable across Nigeria. For example, states, such as Katsina (northern), Cross River (southern), and Niger (western), have rates of unmet need exceeding 20%. Figure 1(IV) illustrates the SDG 3.7.1 indicator demand satisfied evaluated for each Nigerian state, combining mCPR, unmet need, and traditional contraceptive rates. With the exception of Kaduna and Plateau, most northern states have rates of demand satisfied below 20%. The southern regions have higher rates of demand satisfied, but still do not surpass 75%.

The Bayesian hierarchical model produces accompanying uncertainty intervals for each mean estimate. Figure 1(II) illustrates the modeled uncertainty for the estimates in the state Anambra. The posterior median and 95% credible interval (shaded region) show the difference between the modeled estimates with the different survey instruments. Moreover, the model enables short-term forecasting of median estimates with corresponding uncertainty intervals past the most recent survey instrument. A detailed description of the selected models for each indicator is summarized in Additional file 1 (Section 3). See Additional file 1 (Section 5) for visualizations of each PMA2020 state’s temporal history of survey instruments, modeled estimates, and projections to 2018.

### MCPR and unmet need varies by age, parity, and state

**Fig. 2:**
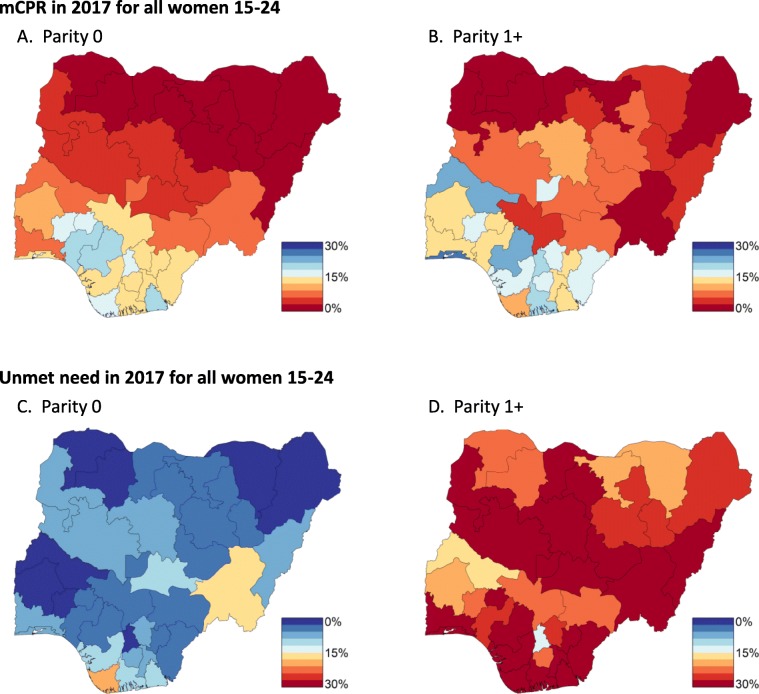
Posterior median estimates of mCPR (top) and unmet need (bottom) by demographic subgroups

State level estimates of unmet need vary significantly between nulliparous and parous women. Figure 2 illustrates uniformly low rates of unmet need for nulliparous women in the 15−24 age cohort. For parous women, however, rates of unmet need vary significantly across the country; for example, Ebonyi, Benue, Cross River, and Bayelsa have rates of unmet need near 30%. The northern states of Nigeria broadly have low rates of mCPR regardless of parity, however, rates of unmet need for parous women are relatively high in the north. Qualitatively, patterns of unmet need and mCPR by state, age, and parity are similar for women older than 25; see Additional file 1 (Section 6) for details. Quantitatively, though, rates of unmet need for parous, older women are uniformly higher than the younger cohort across Nigerian states. Maps of posterior medians of FP indicators by state, age, and parity can be found in Additional file 1 (Section 6).

### Differences across survey instruments in Nigeria

**Fig. 3:**
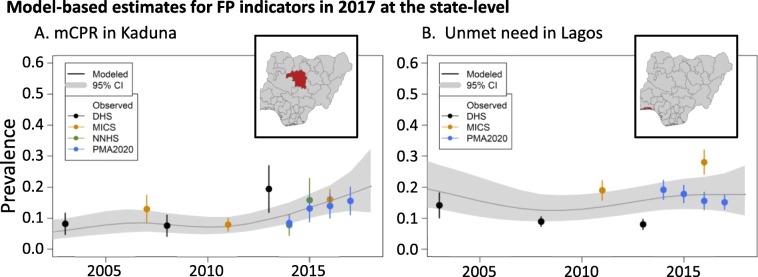
Direct and model-based estimates of mCPR in Kaduna (A) and unmet need in Lagos (B)

Between 70% and 99% of the overall variation is described by the spatial and space-survey random effects for nearly all outcomes and demographic subgroups, suggesting substantial spatial heterogeneity in overall rates and survey measurements; see Additional file 1 (Section 4) for more details. The model for unmet need is an exception: nearly 15% of the variation is described by the survey type random effect, suggesting, on average, MICS observations are highest, DHS are lowest, and PMA2020 are in between. This is clearly illustrated by examining direct survey and model-based estimates of unmet need in Lagos; see Fig. 3(B). A similar trend can be found in Anambra, Kano, Nasarawa, Rivers, and Taraba states; Additional file 1 (Section 5) provides more detail for these states.

### Kaduna and Lagos: a case study in conflicting trends

Kaduna and Lagos state are current targets for large investments through the NURHI. Figure 3 displays the direct and model-based estimates for mCPR in Kaduna (A) and for unmet need in Lagos (B). The 2013 DHS direct estimate of mCPR in Kaduna suggests a large increase relative to the 2011 MICS direct estimate, but given the large uncertainty in the 2013 direct estimate and subsequent mCPR observations, the model estimates a more gradual increase since 2011. In Lagos, direct estimates from different survey instruments suggest conflicting trends in unmet need, with flat trends in DHS, increases in MICS, and a decline observed in PMA2020. Due to this large variability by survey type, the model estimates a flat trend and large uncertainty in the underlying mean. The direct and model-based estimates for all FP indicators in PMA2020 states can be seen in Additional file 1 (Section 5).

### Estimating mCPR changes from 2012 to 2017

**Fig. 4:**
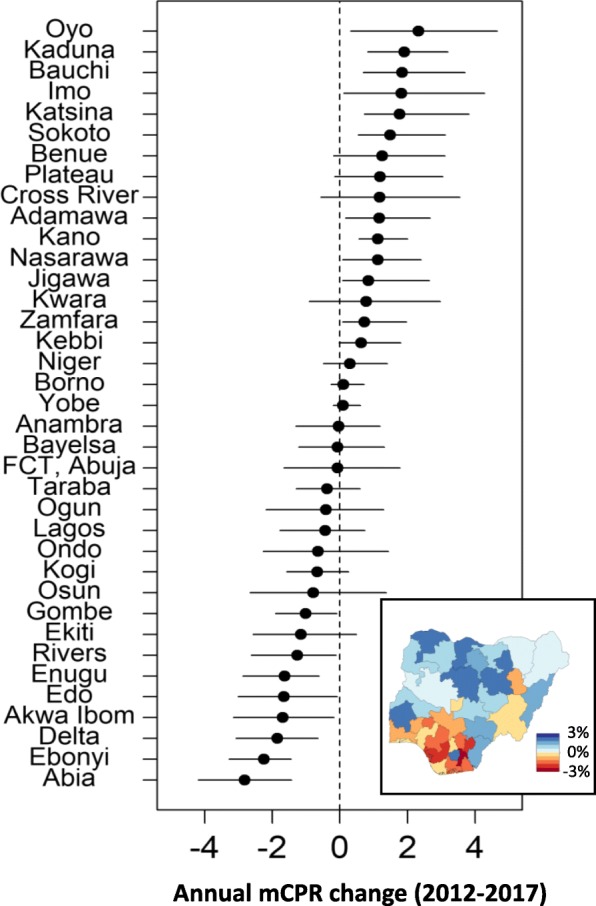
Posterior median and 95% credible interval of annual change in mCPR from 2012 to 2017 and posterior median mCPR in 2017 (inset)

Only eleven states have estimated changes significantly greater than zero. Furthermore, only nineteen out of thirty-six states and the Federal Capital Territory have positive estimates of annual change from 2012 to 2017. Figure 4 displays the posterior median and 95% credible intervals of the annual change in mCPR from 2012 to 2017. Interpretation of annual changes should also consider population increases by state. For example, according to the geo-referenced state level population estimates available for Nigeria (https://nga.geopode.world/), the estimated number of modern contraceptive users in Ondo state is approximately 150,000 women in both 2012 and 2017. Though not statistically significant, the stable number of users corresponds to an estimated 0.6% annual decrease over the five-year period for Ondo state due to population growth. Furthermore, if current state level estimates are applied to the expected population of women between the ages of 15 to 49 in 2030 the total users of modern contraception would grow from approximately 5.0 to 6.7 million women, but overall national estimates would decrease from 11.1% to 10.6% due to larger cohorts of young women in states with lower mCPR.

## Discussion

We have constructed a model to generate estimates for the underlying rates of contraceptive prevalence, unmet need, and demand satisfied at a sub-national resolution for Nigeria. In a principled statistical framework, we have integrated spatial statistics, survey statistics, and multiple measurement instruments to produce sub-national annual estimates and short-term future forecasts. Our statistical methodology is fundamentally data-driven; we incorporate all available survey data from Nigeria, include survey-specific designs and sampling uncertainty, and construct parsimonious, spatio-temporal statistical models for sub-national areas. By utilizing a Bayesian hierarchical model, we can systematically incorporate sampling and non-sampling error for all available surveys. Our approach better characterizes the underlying landscape of FP indicators in Nigeria and enables public health officials to holistically interpret new survey results.

The generation of model-based estimates for FP indicators across Nigerian states revealed significant challenges to interpreting sub-national survey results. For example, the 2013 DHS direct estimate for Kaduna state presented an approximately 10% increase of mCPR over two years. However, in the context of other subsequent surveys, including PMA2020, NNHS, and MICS, the DHS direct estimate appears unlikely; see Fig. 3(A) for reference. One advantage of our Bayesian hierarchical framework is identifying survey specific effects. Figure 3(B) shows three different survey instruments, when taken individually, describe varying temporal trends, i.e., increasing, decreasing and staying uniform. The model-based estimate can integrate each of these estimates and identify systematic differences in survey types by FP indicator, such as relatively high estimates from MICS and relatively low estimates from DHS of unmet need. Without model-based estimates, it would be easy to overinterpret sub-national differences in direct estimates of FP indicators across a variety of survey instruments.

Our modeling framework can be an enabling technology for public health officials and policy-makers in Nigeria. Accounting for available survey data, integrating data into model-based estimates, and quantitatively identifying sources of variation in the data and estimates, we can provide estimates of the true *underlying* mCPR, traditional contraceptive rates, unmet need, and demand at a spatial resolution more aligned with FP programs and interventions. These state level estimates provide public health officials and policy makers a basis to design or interpret the impact of family planning programs or interventions. For example, mCPR is expectedly low in the northern states of Nigeria, generally aligning with other primary health indicators for routine services, such as routine immunization [30]. Rates of unmet need, however, vary significantly across the country providing a more nuanced perspective of family planning indicators within Nigeria; see Fig. 1(III.B) for an illustration. Moreover, summarizing unmet need according to age and parity, we see substantial heterogeneity sub-nationally and by parity. Our approach can help inform the allocation of resources and program implementation, such as post-partum family planning programs in states with high rates of unmet need. In a country as large, diverse, and populous as Nigeria, interventions and programs will be implemented at much smaller spatial scales; using model-based estimates such as the one described in this article could enable better programmatic decisions.

On a broader note, our modeling framework and results are largely consistent with other modeling methodologies and help frame global family planning goals in a more local context. Establishing national baselines in 2012 and tracking yearly progress was essential to characterizing progress toward *120 by 20* [1] in terms of required changes in national mCPR [7]. To date, however, estimated progress toward *120 by 20* is lacking [5] as well as country specific goals [7]. Our sub-national modeling efforts aim to characterize the underlying rates of FP indicators to help identify acceleration opportunities at the spatial scale of programs and interventions. Figure 4 illustrates Nigerian state level annual mCPR changes from 2012-2017; combined with population estimates, the absolute number of new users can be computed for each state. Our results confirm that at the national level mCPR has not changed significantly since 2012. However, our results have also expanded the scope of previous modeling and analyses by identifying which regions have statistically seen increases, decreases, or stayed neutral for mCPR.

Data availability poses serious constraints on our framework being used as a baseline and forecasting tool for small subregions in a country like Nigeria. Despite the variety of survey instruments and known survey cluster locations, estimating FP indicators at the local government area (LGA) spatial. scale produces substantial uncertainty intervals, illustrated by Figure 12 in the Additional file 1. There are also several open questions about constructing the spatio-temporal terms in our Bayesian hierarchical model: what are the relevant geographic regions to include in the spatial smoothing procedure?, which model selection criteria is most appropriate for these models?, and can routine data from the District Health Information Software be integrated and trusted in settings like Nigeria? Moreover, our forecasting methodology, using a data-driven, non-parametric, spatio-temporal model, generates rapidly growing uncertainty intervals over a relatively short three-year outlook.

Notwithstanding these challenges and limitations to our approach, both types of model uncertainty provide an essential result for policy makers and large-scale donors to realistically frame the resolution available to evaluate the impact of investments at a fine spatial scale. We have mitigated a number of the open questions around model construction by building several standard models and utilizing a model selection procedure consistent with research in the small area estimation community for under five mortality estimation [11]. Our model selection and sensitivity analyses can be found in the Additional file 1 which we believe will inform other FP subnational modeling efforts. We also expect to address more of these limitations and challenges in future work.

## Conclusions

Our study can enable public health officials and policy makers through better characterization of FP indicators at a sub-national and demographic subgroup scale. Given the current lag in progress toward *120 by 20*, a change of perspective is broadly required. The FP community should shift from measuring national progress with an assumed broad increase of mCPR to a sub-national perspective focused on informing specific programs at the scale that programs are implemented. Looking forward, the design and implementation of FP programs, funded by governmental and non-governmental organizations, need to have the most holistic and informed estimates to understand the current estimates and trends of FP indicators sub-nationally. The global health community is poised to make significant gains to provide equitable access to family planning services; having a paired measurement and program strategy will be key to achieving these goals.

## Supplementary information

**Additional file 1** Supplementary materials.

## Data Availability

All raw data is available by request through the respective survey websites, DHS (https://dhsprogram.com/), MICs (http://mics.unicef.org/), PMA2020 (https://www.pma2020.org/), and NNHS (https://www.nigerianstat.gov.ng/nada/index.php/home). Intermediate datasets and the R scripts for all analyses will be available on github repository.

## Abbreviations

BMGF: Bill and Melinda Gates Foundation
DHS: Demographic and Health Surveys
DIC: Deviance Information Criteria
DMPA-SC: Depo-medroxyprogesterone Acetate - subcutaneous
FP: Family Planning
FPET: Family Planning Estimation Tool
INLA: Integrated Nested Laplace Approximation
LGA: Local Government Area
LCPO: Log Conditional Predictive Ordinate
mCPR: Modern Contraceptive Prevalence Rate
MCMC: Markov Chain Monte Carlo
MICs: Multiple Indicator Cluster Surveys
NNHS: National Nutrition and Health Surveys
NURHI: Nigerian Urban Reproductive Health Initiative
PMA2020: Performance, Monitoring, and Accountability 2020
SAE: Small Area Estimation
SDG: Sustainable Development Goal
USAID: United States Agency for International Development
WAIC: Watanabe-Akaike Information Criterion
