# Supplementary material for: Sub-national levels and trends in contraceptive prevalence, unmet need, and demand for family planning in Nigeria with survey uncertainty
Source: BMC Public Health. 2019 Dec 30;19:1752. doi: 10.1186/s12889-019-8043-z (PMC6937659; doi:10.1186/s12889-019-8043-z)
Supplement: Supplementary file 1 — Additional file 1 Supplementary materials. [file 12889_2019_8043_MOESM1_ESM.pdf]

Supplement for ‘Sub-national levels and trends in contraceptive prevalence, unmet need, and demand for family planning in Nigeria with survey uncertainty’

Laina D. Mercer, Fred Lu, and Joshua L. Proctor

Institute for Disease Modeling
Bellevue, WA, 98005

1. Input Data

In this section we describe the thirteen household surveys that were analyzed for the article ‘Sub-national levels and trends in contraceptive prevalence, unmet need, and demand for family planning in Nigeria with survey uncertainty.’

1.1. Demographic and Health Surveys

We processed the four available Demographic and Health Surveys (DHS) for Nigeria. The 1990 DHS selected 34 households from each of the 299 enumeration areas (EAs), which were stratified by urban/rural status and selected via probability proportional to size (pps), and was designed to produce national and regional (4 regions at that time) estimates [1]. A stratified two-stage sampling design was used for the 2003 DHS to provide estimates of health indicators at the national level, the six health regions, and by urban/rural status. Households were select from a complete listing of households within the 365 selected EAs [2]. Similarly, the 2008 and 2013 DHS employed stratified multi-stage sampling designs consisting of 888 EAs and 904 EAs, respectively [3, 4] and were intended to be representative at the national, regional, and state level. An additional survey was conducted in 1999, however the DHS Program was not centrally involved with the study and does not distribute the collected data.

To calculate unmet need we relied on the recoded definitions used by the United Nations Population Division (UNPD), which applied the revised DHS unmet need definition [5] to previous surveys. To compute the revised unmet need in our analysis, we relied on the UNPD script available at `https://github.com/PhilUeff/PDU_FP-Indicators`.

To ensure state-level values reflect current geographical divisions, we mapped the GPS coordinates associated with the sampled clusters from each survey onto the 2013 Nigeria DHS shapefile and used their corresponding state names. Each DHS survey contained a few clusters without recorded GPS coordinates (Table 1). For these, we assigned the clusters to the states that they were recorded as belonging to at the time. The assignment is ambiguous for one DHS 1990 cluster because the corresponding recorded state has split between the time of the survey and 2013; this is noted in Table 1.

Table 2 compares national all-women estimates for contraceptive prevalence rate (CPR), modern contraceptive prevalence rate (mCPR), total unmet need (UnN), and demand satisfied by modern methods (DSM), as computed by the DHS survey official reports, the UN recode, and our analysis. While not presented, traditional contraceptive prevalence rate can be computed as CPR - mCPR.

Contraceptive prevalences match among all sources. The differences in unmet need between the DHS official reports and the UN computed values prior to 2012 reflect the revised unmet need calculation. Dashes indicate estimates that were not reported by the DHS.

Table 1: Surveyed DHS clusters without GPS coordinates

DHS year	Cluster ID	Recorded state	Possible states
1990	1402	Kwara	Kwara, Kogi
	1554	Lagos	Lagos
2003	170	Katsina	Katsina
	258	Lagos	Lagos
2008	773	Rivers	Rivers
2013	302	Yobe	Yobe
	373	Kano	Kano
	422	Kebbi	Kebbi
	514	Anambra	Anambra
	557	Enugu	Enugu
	569	Enugu	Enugu
	639	Bayelsa	Bayelsa

1.2. Multiple Indicator Cluster Surveys

We used three Multiple Indicator Cluster Surveys (MICS) surveys in our analysis. Thirty EAs were selected within each state for the 2007 MICS, which was designed to provide state and national level estimates of health indicators [6]. A two-stage sampling design was used for the 2011 MICS, which was designed to provide estimates and the state and national level.

Table 2: DHS surveys: comparing DHS reported figures, UN computed figures, and our computed figures at the national level for all women. All values are reported in (%).

	DHS reported				UN computed				Our computed			
DHS year	CPR	mCPR	UnN	DSM	CPR	mCPR	UnN	DSM	CPR	mCPR	UnN	DSM
2013	16.0	11.1	12.7	38.8	16.0	11.1	12.7	38.5	16.0	11.1	12.7	38.8
2008	15.4	10.5	15.7	–	15.4	10.5	16.1	33.2	15.4	10.5	16.1	33.2
2003	13.3	8.9	13.6	–	13.3	8.9	14.6	32.0	13.3	8.9	14.6	32.0
1990	7.5	3.8	–	–	7.5	3.8	17.1	15.2	7.5	3.8	17.1	15.2

Forty EAs were selected with equal probability within each state and all 1,480 EAs were included in field work [7]. The 2016-17 MICS was designed to provide estimates at the national and state level and relied on a two-stage sampling design. Sixty EAs were selected from each state, except for Lagos and Kano which were over sampled at 120 EAs. Of the 2,340 sampled EAs only 2,239 were included in field work because 101 EAs from Borno, Yobe, and Adamawa states were excluded due to security concerns [8].

The UNPD has recoded the 2007 and 2011 MICS surveys, but at the time of this writing, had not yet recoded the 2016 survey. We used the UNPD recode scripts from `https://github.com/PhilUeff/PDU_FP-Indicators` to process the 2007 and 2011 MICS survey data, then computed the survey estimates as with the DHS surveys. When we used the UN code on the data, we found a small difference in the 2011 estimated unmet need from the value reported by the UNPD. For the 2016 MICS survey, we checked that the variables were encoded in the same form as the 2011 MICS survey. The unavailable estimates from the MICS official reports and 2016 UNPD recode are indicated with dashes. Finally, certain questions needed to calculate unmet need were not asked in the 2007 MICS survey. As a result, unmet need and demand satisfied for this survey are marked with 'NA'. The MICS official reports only present estimates over currently-married/in-union women. Table 3 displays the estimates reported by MICS, calculated by the UN, and calculated via our direct analysis of the microdata.

1.3. Performance Monitoring and Accountability 2020

We used four rounds of the Performance Monitoring and Accountability 2020 (PMA2020) surveys conducted in Nigeria. We obtained the data for the 2014-2016 PMA2020 rounds [9, 10, 11] from IPUMS [12], which had recoded the PMA2020 surveys. We computed survey estimates using this data. The 2017 PMA2020 survey [13] had not been recoded at the time of this writing by IPUMS, so this survey was recoded manually. The 2014 and 2015 rounds were designed to provide state level estimates for Kaduna and Lagos states and relied on a two-stage sampling design. The 2016 and 2017 rounds were designed to provide national estimates relying on three-stage sampling design. At the first stage seven states were selected and then within each stage two-stage sampling was implemented. Post stratification was used to generate national estimates [14]. Table 4 provides the point estimates reported by PMA2020 and those calcuated in our analysis of the microdata.

1.4. National Nutrion and Health Surveys

We analyzed the microdata from two National Nutrition and Health Surveys (NNHS) from 2014 and 2015. Both surveys were conducted using the Standardized Monitoring and Assessment of Relief and Transition (SMART) methods and relied on two-stage cluster and designed to provide estimates at the national and state level [15, 16]. Unfortunately, the NNHS does not include the variables required to calculate unmet need nor parity, so the NNHS data was only included in the estimates of mCPR for all women. Additionally, the survey reports highlight that in 9 Local Governmental Areas (LGAs) were excluded for security reasons and thus results for Borno are not representative of the whole state.

2. Modeling family planning indicators

Family planning indicators in Nigeria are estimated from multiple complex surveys, sometimes in the same year and can be quite noisy between surveys at the state level. In an effort to understand the underlying population rates from which these survey observations were drawn, we are adapting a previously developed two-step space-time smoothing approach that acknowledge complex sampling designs to model the family planning indicators.

The first step of our approach requires estimating the state (i), year (t), and survey (s) specific direct estimates of proportions and corresponding variance via the Hajek estimator [17] $\widehat{p}_{its} = \sum_j x_{j,its} w_{j,its} / \sum_j w_{j,its}$ where $x_{j,its}$ is the binary indicator for modern contraception, traditional contraception, unment need, or demand satisfied for woman j, in sampled in area i, time t, and survey s, and $w_{j,its}$ is her corresponding sampling weight.

In the second step we use a three-stage Bayesian hierarchichal model. In the first stage we rely on the working likelihood based on the asymptotic distribution,

$$Y_{its} \sim N\left(\eta_{\mathbf{its}}, \widehat{V}_{its,DES}\right)$$

Table 3: MICS surveys: comparing MICS reported figures, UN computed figures, and our computed figures at the national level for all women. All values are reported in (%).

	MICS reported				UN computed				Our computed			
MICS year	CPR	MCPR	UnN	DSM	CPR	MCPR	UnN	DSM	CPR	MCPR	UnN	DSM
2016	–	–	–	–	–	–	–	–	11.8	9.5	26.8	24.6
2011	–	–	–	–	18.3	12.5	20.2	32.6	18.3	12.5	21.0	31.9
2007	–	–	NA	NA	14.6	11.1	NA	NA	14.6	11.1	NA	NA

Table 4: PMA surveys: comparing PMA2020 reported figures and our computed figures at the national level for all women. All values are reported in (%).

		PMA2020 reported				Our computed			
PMA year	State	CPR	MCPR	UnN	DS	CPR	MCPR	UnN	DS
2017	Anambra	24.2	13.9	12.0	38.3	24.1	13.8	12.0	38.2
	Kaduna	17.4	15.6	22.4	39.2	17.4	15.6	22.4	39.2
	Kano	6.2	4.4	24.6	14.4	6.2	4.4	24.6	14.4
	Lagos	29.7	20.6	15.2	45.8	29.7	20.6	15.2	45.8
	Nasarawa	18.9	16.9	21.0	42.3	18.9	16.9	21.0	42.3
	Rivers	29.0	17.7	17.2	38.2	29.0	17.7	17.2	38.2
	Taraba	14.1	10.5	24.4	27.3	14.1	10.5	24.4	27.3
2016	Anambra	25.1	15.6	14.4	39.6	25.9	16.1	14.4	40.4
	Kaduna	15.1	13.9	26.2	33.7	15.4	14.1	26.2	34.0
	Kano	5.6	4.8	30.3	13.5	5.6	4.9	30.2	13.5
	Lagos	26.4	19.7	15.6	46.8	26.0	19.7	15.6	46.9
	Nasarawa	18.9	16.6	18.0	44.9	18.9	16.6	18.0	44.9
	Rivers	27.5	19.4	16.4	44.2	28.8	19.6	16.4	43.7
	Taraba	12.9	9.9	27.1	24.9	12.6	10.0	27.1	25.0
2015	Kaduna	14.5	13.2	24.9	33.5	14.7	13.5	24.9	34.2
	Lagos	27.8	21.0	17.8	46.1	27.8	21.4	17.8	46.4
2014	Kaduna	8.7	8.4	28.2	22.8	8.7	8.5	28.2	22.9
	Lagos	17.8	16.5	19.3	44.5	17.3	16.7	19.2	44.8

where $Y_{its} = \text{logit}[\widehat{p}_{its}]$ and $\widehat{V}_{its,DES}$ is the design-based variance of Y_{its}. This pseudo-likelihood for a binary variable was initially developed to estimate zipcode-level smoking prevalence in Washington state [18] relying on a single survey instrument. This model was then adapted for the complex indicator of under-five child mortality [19] and extended the classic inseperable spatio-time models of [20, 21] to include survey-specific random effects.

At the second stage we assume

$$\eta_{its} = \mu + \gamma_t + \alpha_t + \theta_i + \nu_s + \delta_{it} + \phi_{is} + \psi_{ts},$$

where μ is a shared interecept, the temporally structured random effects α are assigned second order random walk priors [22, 21], the spatial random effects θ are assigned either the Besag, York, and Mollie (BYM) [23] or the scaled BYM [24], the space-time interactions δ are assinged the 'type II' temporally structured interaction [21], and the independent random effects

γ, ν, ϕ, and ψ are assigned independent mean-zero Normal distributions. Note, if θ ~BYM then $\theta_i = U_i + V_i$ where U is assigned an intrinsic conditional autoregression prior (ICAR) [25] and V are assigned an independent mean-zero Normal prior. At the third stage of the model we assume a default diffuse prior for μ and Gamma$(1, 5e-5)$ priors were assigned to, σ_α^{-2}, σ_θ^{-2} (or to both inverse variances for standard BYM), σ_γ^{-2}, σ_δ^{-2}, σ_ν^{-2}, σ_ϕ^{-2}, and σ_ψ^{-2}.

Finally, to generate our estimates of the underlying rates we adopt the approach of Mercer (2015) and draw k=1,...1000 posterior samples for all parameters and calculate

$$\eta_{it}^{(k)} = \mu^{(k)} + \gamma_t^{(k)} + \alpha_t^{(k)} + \theta_i^{(k)} + \delta_{it}^{(k)}.$$

The posterior median is used as the estimate and the uncertainty intervals are defined by the 2.5% and 97.5% quantiles.

3. Model Selection

The twelve possible models considered for mCPR, traditional contraceptive prevalence, unmet need, and demand satisfied are described in Table 5. Each model was fit for mCPR, traditional contraceptive prevalence, unment need and demand satisfied for all women and the four age and parity combinations. For each model fit we calculated the sum of the log conditional predictive ordinate (LCPO) [26], the deviance information criteria (DIC) [27], and the Watanabe-Akaike information criterion (WAIC) [28]. The results for mCPR and unment need for all women are shown in Table 6 and Table 7, respectively with with the lowest DIC and WAIC and highest LCPO in bold. Table 8 shows the selected model for all outcomes and age-parity groups.

Table 5: Random effects models considered for time t, state i, and survey s. All models contain an intercept μ, temporally independent $\gamma_t \sim N(0, \sigma_\gamma^2)$, and temporally structured effect $\alpha_t \sim RW2(\sigma_\alpha^2)$. The survey and survey interaction random effects were assigned mean-zero Normal priors. In models designated with 'a' $\theta_i \sim BYM(\sigma_\theta^2)$ and in models designated 'b' $\theta_i \sim BYM2(\sigma_\theta^2)$.

Model	outcome
1	$\mu + \alpha_t + \gamma_t + \theta_i$
2	$\mu + \alpha_t + \gamma_t + \theta_i + \delta_{it}$
3	$\mu + \alpha_t + \gamma_t + \theta_i + \delta_{it} + \nu_s$
4	$\mu + \alpha_t + \gamma_t + \theta_i + \delta_{it} + \nu_s + \phi_{is}$
5	$\mu + \alpha_t + \gamma_t + \theta_i + \delta_{it} + \nu_s + \psi_{ts}$
6	$\mu + \alpha_t + \gamma_t + \theta_i + \delta_{it} + \nu_s + \phi_{is} + \psi_{ts}$

4. Decomposition of Variance

To assess the relative importance of the individual random effects in explaining the variance in the observed data we calculated the percent of the total variance described by each random effect. Table 9 shows the percent of the variance described by each random effect where the total variance for model 6 is

$$\sigma_\alpha^2 + \sigma_\gamma^2 + \sigma_\theta^2 + \sigma_\delta^2 + \sigma_\nu^2 + \sigma_\psi^2 + \sigma_\phi^2$$

and for outcomes and age-parity groups using model 4, the σ_ϕ^2 term is removed.

Table 6: Model fit results for estimating mCPR for all women.

Model	DIC	$\sum \log(CPO)$	WAIC
1a	1418.1	-846.0	1663.6
2a	514.7	-535.9	697.6
3a	483.7	-525.9	658.4
4a	419.2	-515.0	568.8
5a	477.6	-507.6	643.7
6a	402.7	-490.5	539.6
1b	1418.1	-845.8	1663.3
2b	514.3	-535.4	695.7
3b	483.2	-525.1	655.9
4b	418.6	-513.5	567.7
5b	476.6	-506.9	641.9
6b	**402.1**	**-488.5**	**538.4**

Table 7: Model fit results for estimating unmet need for all women.

Model	DIC	$\sum \log(CPO)$	WAIC
1a	1145.3	-748.4	1487.7
2a	27.6	-298.9	90.7
3a	5.0	-283.3	58.8
4a	-56.7	-208.5	-56.1
5a	4.4	-281.4	56.7
6a	**-56.8**	**-208.0**	**-56.4**
1b	1145.4	-748.2	1487.3
2b	26.3	-299.3	88.8
3b	3.8	-283.2	56.9
4b	-56.4	-209.5	-55.3
5b	4.2	-281.8	56.0
6b	-56.5	-208.9	-55.7

Table 8: Selected models.

Age-parity group	outcome	selected model
All women	mCPR	6b
	Unmet Need	6a
	tCPR	4b
	Demand Satisfied	6b
15-24yo, 0	mCPR	6a
	Unmet Need	4a
	tCPR	6a
	Demand Satisfied	6a
15-24yo, 1+	mCPR	6b
	Unmet Need	4b
	tCPR	6b
	Demand Satisfied	6a
25-49yo, 0	mCPR	6b
	Unmet Need	6b
	tCPR	6b
	Demand Satisfied	6a
25-49yo, 1+	mCPR	6b
	Unmet Need	6b
	tCPR	4b
	Demand Satisfied	4b

5. Trends in family planning indicators in PMA2020 states

The observed data and smoothed estimates for mCPR, traditional contraceptive prevalence, unment need, and demand satistified in the seven states (Anambra, Kaduna, Kano, Lagos, Nasarawa, Rivers, and Taraba) are shown in Figures 1 through 7.

6. Maps of family planning indicators for all age-parity subgroups

Figures 8, 9, 10, and 11 show the 2017 state-level estimates or the four age-parity subgroups for mCPR, unmet need, traditional contraceptive prevalence, and demand satisfied, respectively.

7. Small area estimation at the second administrative level

The health policies are often enacted at the second adminstrative level, called local government areas (LGAs) in Nigeria, and ideally we would provide estimates at the LGA-level. Unfortunately, MICS, DHS, PMA2020, and NNHS do not inlcude the LGA locations for the sampled clusters. However, the DHS have provided jittered [29] GPS locations which can be assigned LGA membership. In this section we investigate and compare the precision attained at the state- and LGA-level. We apply the methods described in Section 2, removing the survey-related random effects, to the DHS data from 1990. 2003, 2008, and 2013.

The left panel of Figure 12 display the state-level estimates and 95% credible intervals for mCPR and the right panel displays the same, but at the LGA-level. From the figure it is clear that in many of the LGAs the uncertainty is intolerably large. This is not a surprising result, as many LGAs have few or no sampled clusters. By contrast differences between states and within states over time can be reliably estimated; see Figure 12 in the Appendix and Figure 4 in the main article, respectively. With currently available data it is not possible to generate sub-state level estimates for FP indicators with reasonable precision. FP estimates at the LGA rely exclusively on DHS data and are thus not timely nor precise.

References

[1] Federal Office of Statistics/Nigeria and Institute for Resource Development - IRD/Macro International, Nigeria Demographic and Health Survey 1990, Federal Office of Statistics/Nigeria and IRD/Macro International, 1992.

[2] National Population Commission - NPC/Nigeria and ORC Macro., Nigeria Demographic and Health Survey 2003, NPC/Nigeria and ORC Macro, 2004.

[3] National Population Commission - NPC/Nigeria and ICF Macro, Nigeria Demographic and Health Survey 2008, NPC/Nigeria and ICF Macro., 2004.

[4] I. Macro, N. P. Commission, et al., Nigeria demographic and health survey 2013 (2014).

[5] S. E. Bradley, T. N. Croft, J. D. Fishel, C. F. Westoff, Revising unmet need for family planning. (2012).

[6] National Bureau of Statistics (NBS), Nigeria Multiple Indicator Cluster Survey 2007 Final Report, 2007.

[7] National Bureau of Statistics (NBS) and United Nations Children's Fund (UNICEF), Monitoring the situation of children and women, Multiple Indicator Cluster Survey 2011, 2013.

[8] National Bureau of Statistics (NBS) and United Nations Children's Fund (UNICEF), 2017 Multiple Indicator Cluster Survey 2016-17, Survey Findings Report, 2017.

[9] Centre for Population and Reproductive Health (CPRH) University of Ibadan and Centre for Research Evaluation Resources and Development (CRERD) and Population and Reproductive Health Program (PRHP) Obafemi Awolowo University (OAU), Performance Monitoring and Accountability 2020 (PMA2020) Project 2014 Nigeria (2014).

[10] Centre for Research, Evaluation Resources and Development (CRERD), Bayero University Kano (BUK), Performance Monitoring and Accountability 2020 (PMA2020) Project, 2015 Nigeria (2015).

[11] Centre for Research, Evaluation Resources and Development (CRERD), Bayero University Kano (BUK), Performance Monitoring and Accountability 2020 (PMA2020) Project, 2016 Nigeria (2016).

[12] E. H. Boyle, D. Kristiansen, M. Sobek, IPUMS-PMA: Version 2.0 Nigeria (2018).

[13] Centre for Research, Evaluation Resources and Development (CRERD), Bayero University Kano (BUK), Performance Monitoring and Accountability 2020 (PMA2020) Project, 2017 Nigeria (2017).

[14] PMA2020, PMA2020 Household and Female Survey Sampling Strategy in Nigeria (2017).

Table 9: Percent of total variance described by each random effect, where the interpretation is σ^2_θ for spatial, σ^2_γ for independent temporal, σ^2_α for structured (RW2) temporal, σ^2_δ for space-time interaction, σ^2_ν for surveys, σ^2_ϕ for survey-space interactions, and σ^2_ψ for survey-time interaction.

Age-parity group	outcome	σ^2_θ	σ^2_γ	σ^2_α	σ^2_δ	σ^2_ν	σ^2_ϕ	σ^2_ψ
All women	mCPR	74.5	<0.05	<0.05	0.5	<0.05	23.8	1.2
	Unmet Need	<0.05	<0.05	<0.05	0.6	14.8	79.3	5.3
	tCPR	62.2	<0.05	12.5	0.6	<0.05	24.7	-
	Demand Satisfied	43.4	<0.05	<0.05	0.2	<0.05	55.7	0.7
15-24yo, 0	mCPR	97.5	<0.05	<0.05	<0.05	<0.05	2.0	0.5
	Unmet Need	<0.05	<0.05	7.1	<0.05	<0.05	92.9	-
	tCPR	<0.05	<0.05	<0.05	0.1	<0.05	99.9	<0.05
	Demand Satisfied	<0.05	<0.05	<0.05	<0.05	<0.05	93.0	7.0
15-24yo, 1+	mCPR	79.1	<0.05	<0.05	<0.05	<0.05	20.0	0.8
	Unmet Need	56.5	<0.05	24.3	<0.05	<0.05	19.2	-
	tCPR	80.3	<0.05	<0.05	<0.05	<0.05	19.7	<0.05
	Demand Satisfied	<0.05	<0.05	<0.05	<0.05	<0.05	99.9	<0.05
25-49yo, 0	mCPR	69.1	<0.05	2.8	<0.05	<0.05	28.2	<0.05
	Unmet Need	<0.05	<0.05	<0.05	<0.05	<0.05	32.7	67.2
	tCPR	99.9	<0.05	<0.05	<0.05	<0.05	<0.05	
	Demand Satisfied	<0.05	<0.05	<0.05	<0.05	<0.05	99.9	<0.05
25-49yo, 1+	mCPR	97.7	<0.05	<0.05	0.1	<0.05	2.1	0.1
	Unmet Need	1.4	<0.05	<0.05	0.3	19.8	70.1	8.4
	tCPR	62.1	<0.05	13.6	0.3	0	24.0	-
	Demand Satisfied	46.2	<0.05	0.1	0	0	53.6	-

[15] National Bureau of Statistics and UNICEF, Report on the Nutrition and Health Situation of Nigeria, 2014, 2014.

[16] National Bureau of Statistics, Report on the Nutrition and Health Situation of Nigeria, 2015, 2015.

[17] J. Hájek, Discussion of Basu, in: V. Godambe, D. Sprott (Eds.), Foundations of Statistical Inference, Holt, Rinehart & Winston, 1971.

[18] L. Mercer, J. Wakefield, C. Chen, T. Lumley, A comparison of spatial smoothing methods for small area estimation with sampling weights, Spatial Statistics 8 (2014) 69–85.

[19] L. D. Mercer, J. Wakefield, A. Pantazis, A. M. Lutambi, H. Masanja, S. Clark, Space–time smoothing of complex survey data: Small area estimation for child mortality, The Annals of Applied Statistics 9 (2015) 1889–1905.

[20] L. Knorr-Held, Bayesian modelling of inseparable space-time variation in disease risk, Statistics in Medicine 19 (2000) 2555–2567.

[21] B. Schrödle, L. Held, Spatio-temporal disease mapping using inla, Environmetrics 22 (2011) 725–734.

[22] H. Rue, L. Held, Gaussian Markov Random Fields: Theory and Application, Chapman and Hall/CRC Press, Boca Raton, 2005.

[23] J. Besag, J. York, A. Mollié, Bayesian image restoration with two applications in spatial statistics, Annals of the Institute of Statistics and Mathematics 43 (1991) 1–59.

[24] A. Riebler, S. H. Sørbye, D. Simpson, H. Rue, An intuitive bayesian spatial model for disease mapping that accounts for scaling, Statistical methods in medical research 25 (2016) 1145–1165.

[25] J. Besag, C. Kooperberg, On conditional and intrinsic auto-regressions., Biometrika 82 (1995) 733–746.

[26] L. Held, B. Schrödle, H. Rue, Posterior and cross-validatory predictive checks: a comparison of mcmc and inla, in: Statistical modelling and regression structures, Springer, 2010, pp. 91–110.

[27] D. J. Spiegelhalter, N. G. Best, B. P. Carlin, A. Linde, The deviance information criterion: 12 years on, Journal of the Royal Statistical Society: Series B (Statistical Methodology) 76 (2014) 485–493.

[28] S. Watanabe, Asymptotic equivalence of bayes cross validation and widely applicable information criterion in singular learning theory, Journal of Machine Learning Research 11 (2010) 3571–3594.

[29] C. Burgert, B. Zachary, Incorporating geographic information into demographic and health surveys: a field guide to gps data collection. (2011).

[30] Global Administrative Areas , GADM database of Global Administrative Areas, version 3.6. [online] URL: www.gadm.org, 2018.

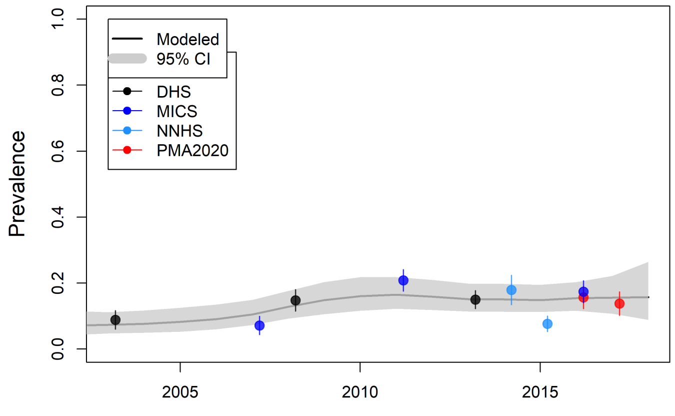

(a) mCPR

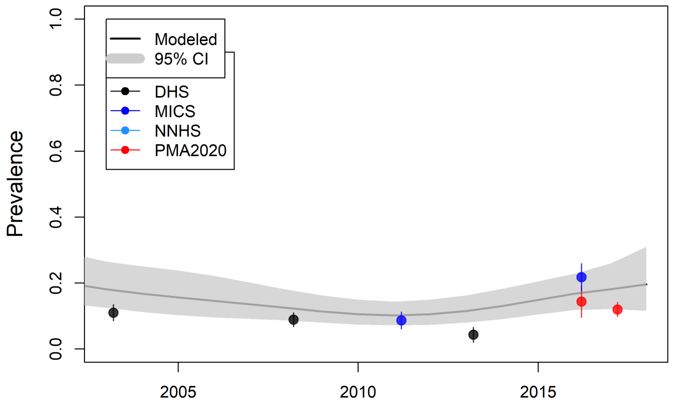

(b) Unmet Need

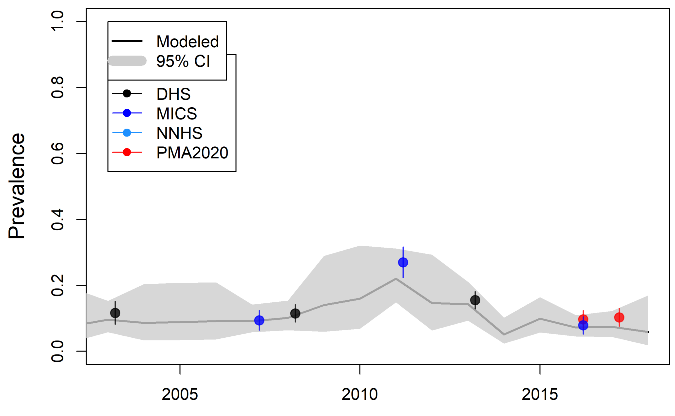

(c) Traditional CPR

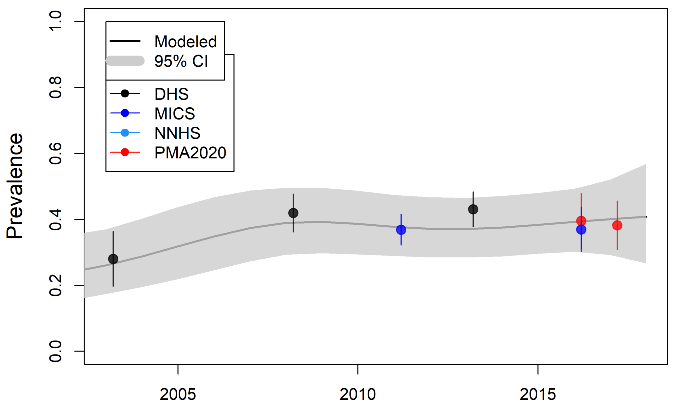

(d) Demand Satisfied

Figure 1: Data and smoothed estimates for family planning indicators in Anambra state.

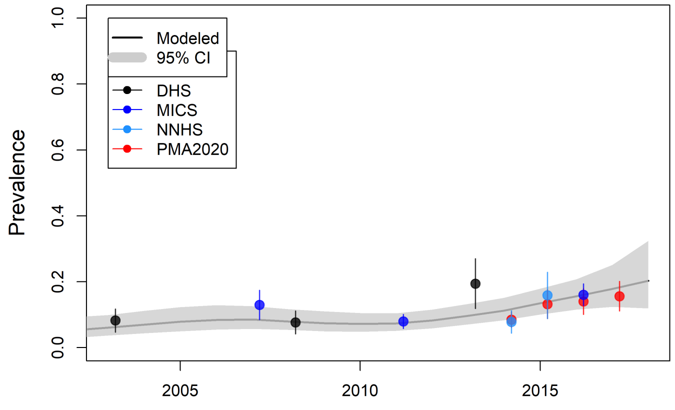

(a) mCPR

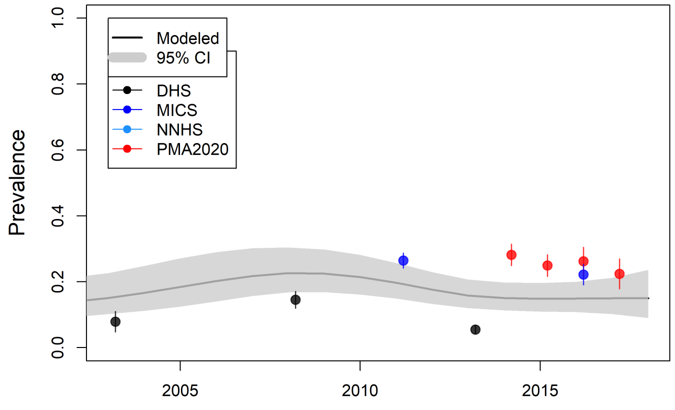

(b) Unmet Need

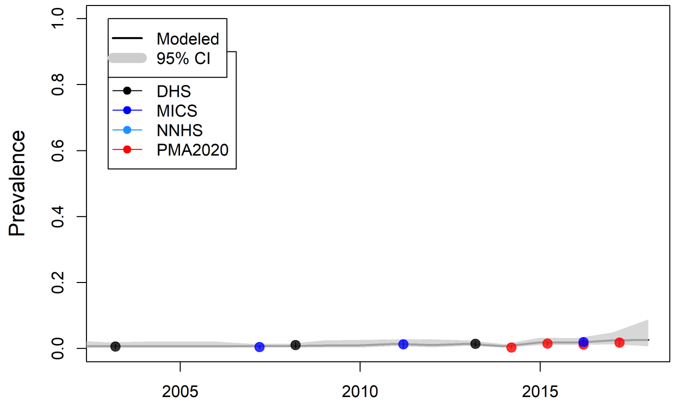

(c) Traditional CPR

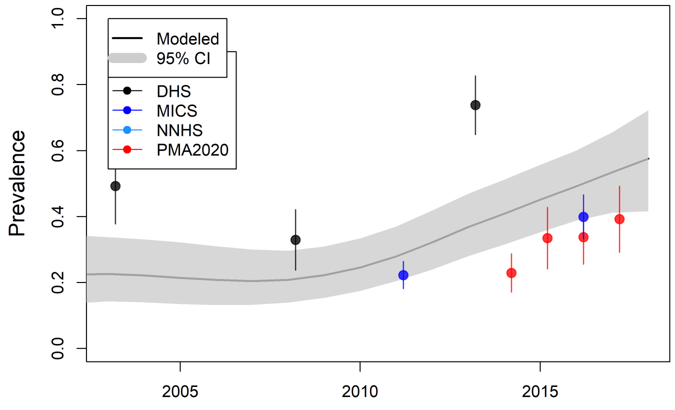

(d) Demand Satisfied

Figure 2: Data and smoothed estimates for family planning indicators in Kaduna state.

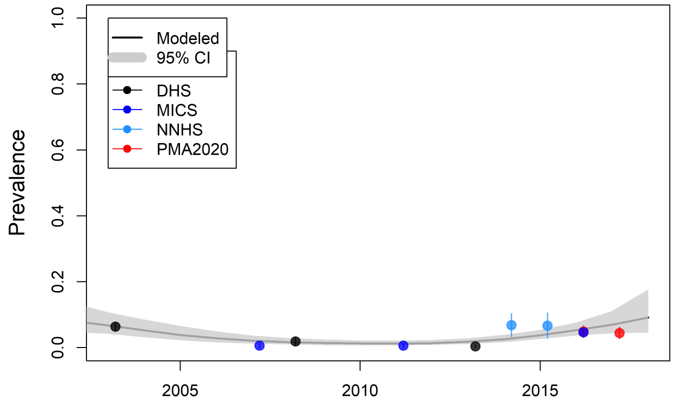

(a) mCPR

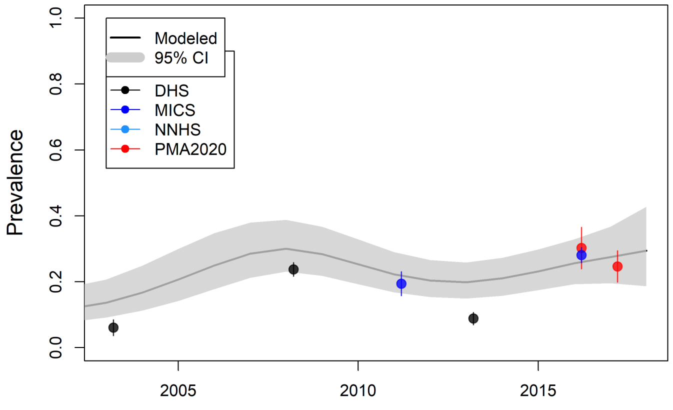

(b) Unmet Need

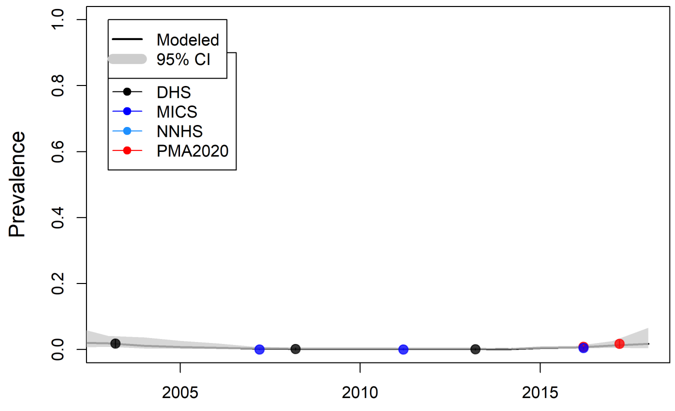

(c) Traditional CPR

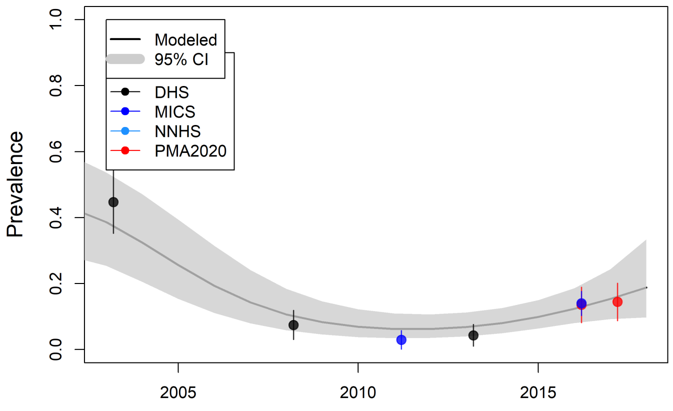

(d) Demand Satisfied

Figure 3: Data and smoothed estimates for family planning indicators in Kano state.

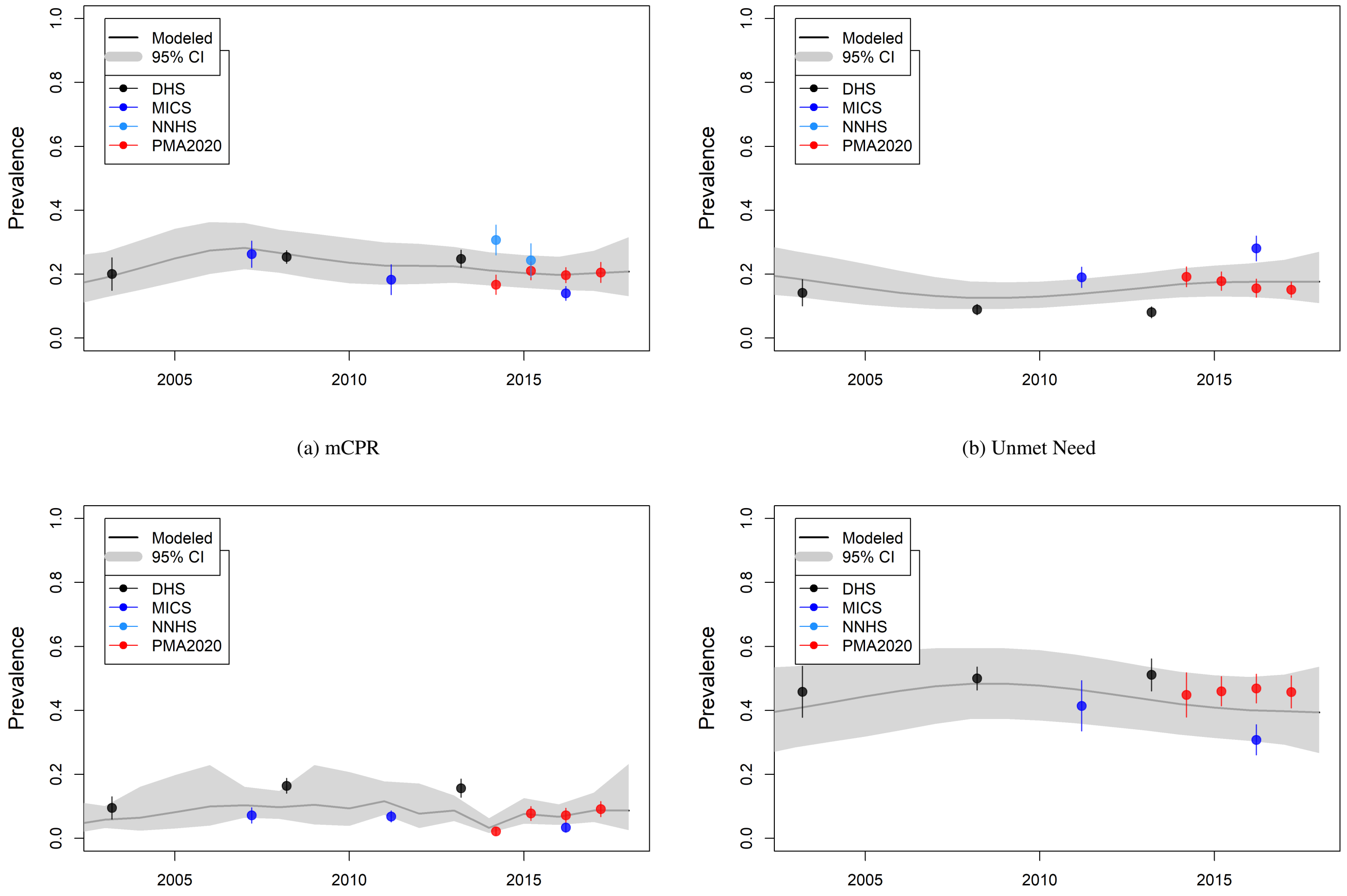

(a) mCPR

(b) Unmet Need

(c) Traditional CPR

(d) Demand Satisfied

Figure 4: Data and smoothed estimates for family planning indicators in Lagos state.

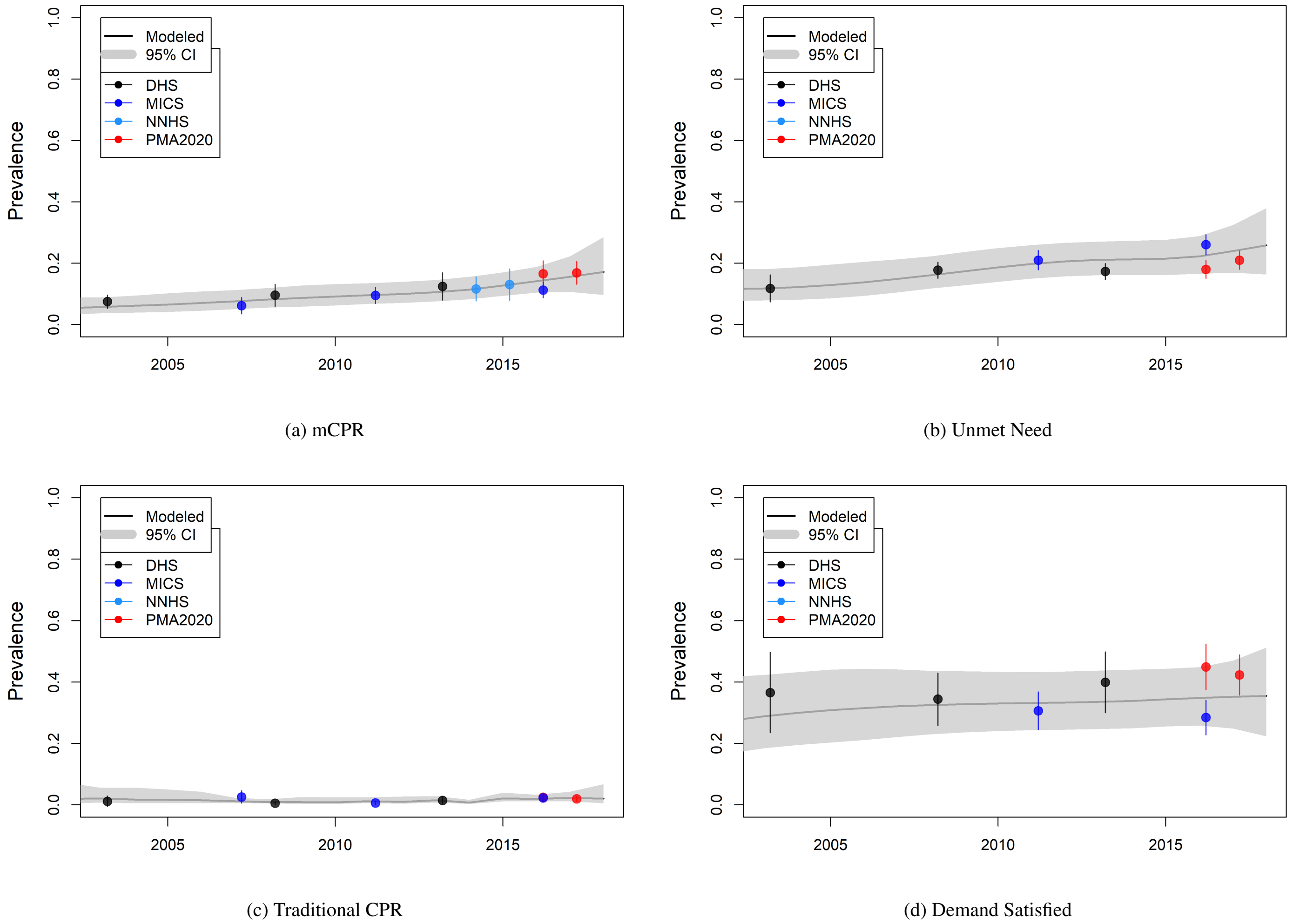

(a) mCPR

(b) Unmet Need

(c) Traditional CPR

(d) Demand Satisfied

Figure 5: Data and smoothed estimates for family planning indicators in Nasarawa state.

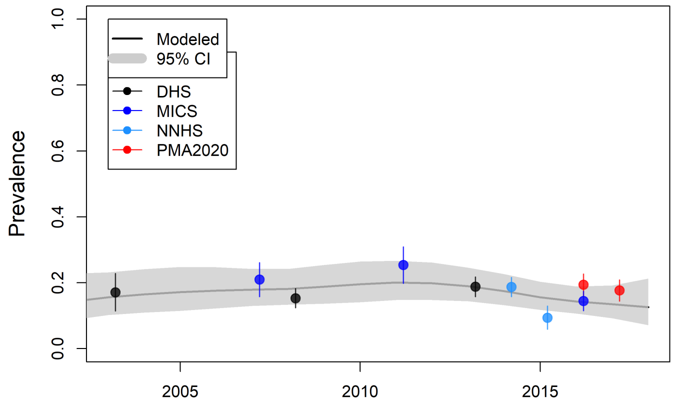

(a) mCPR

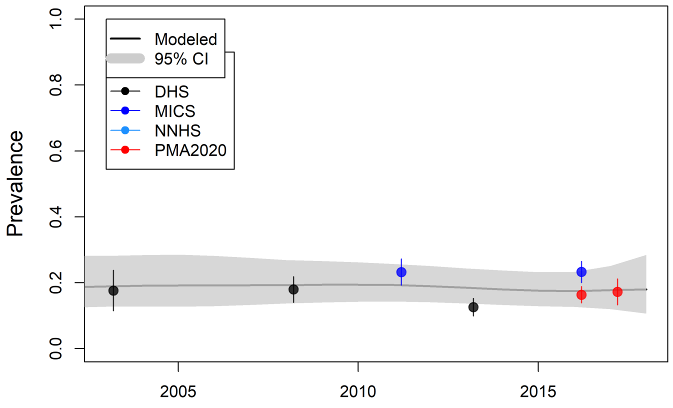

(b) Unmet Need

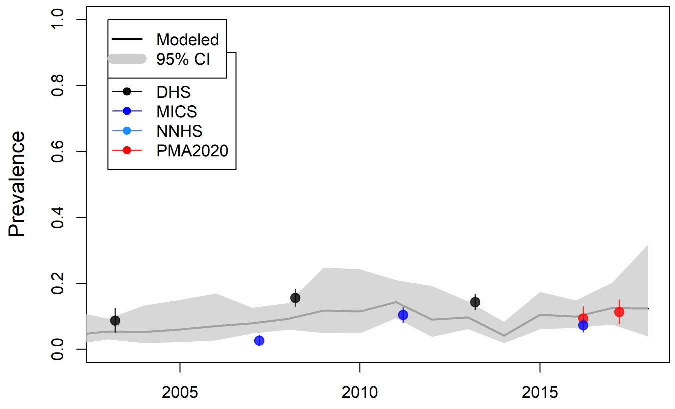

(c) Traditional CPR

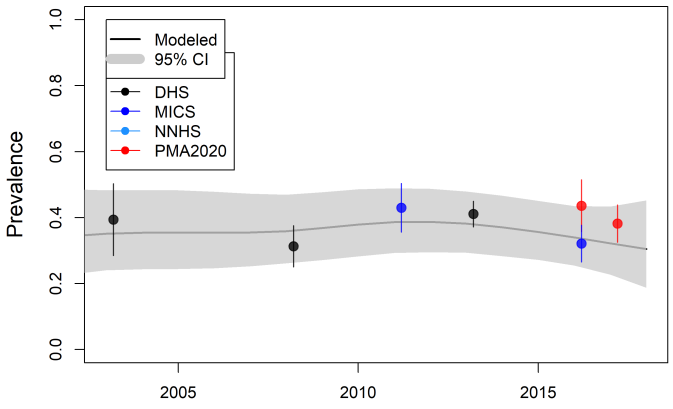

(d) Demand Satisfied

Figure 6: Data and smoothed estimates for family planning indicators in Rivers state.

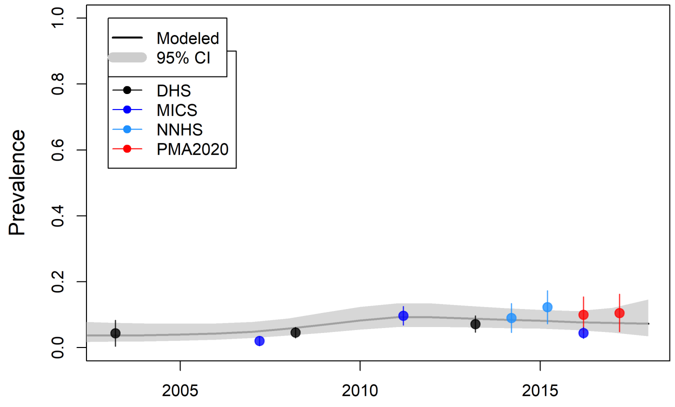

(a) mCPR

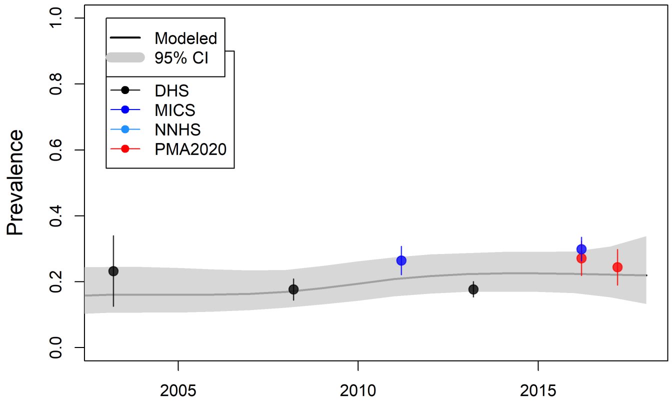

(b) Unmet Need

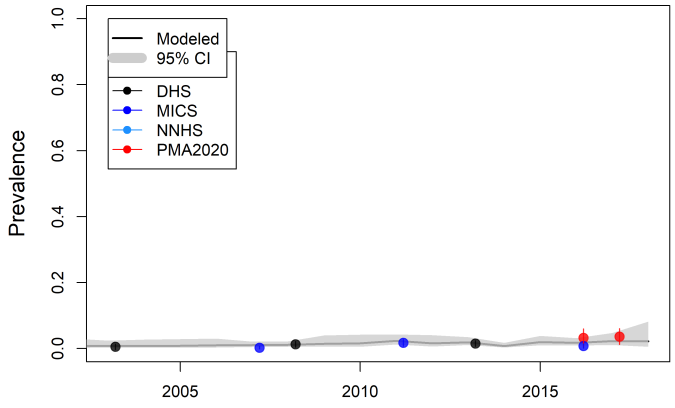

(c) Traditional CPR

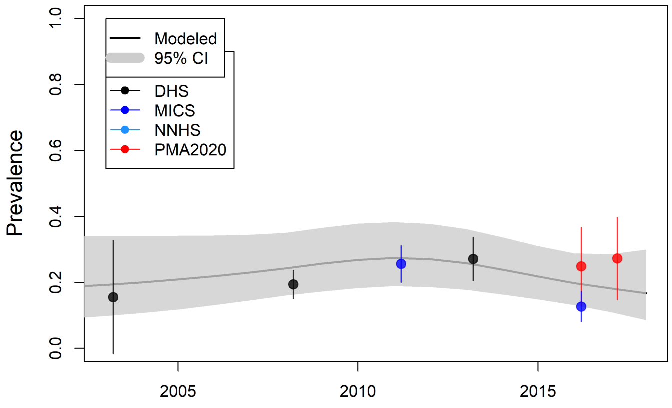

(d) Demand Satisfied

Figure 7: Data and smoothed estimates for family planning indicators in Taraba state.

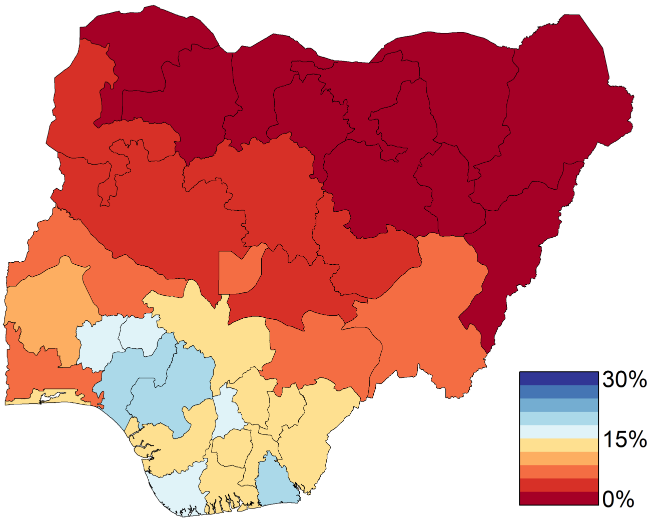

(a) Age 15-24 years and parity 0.

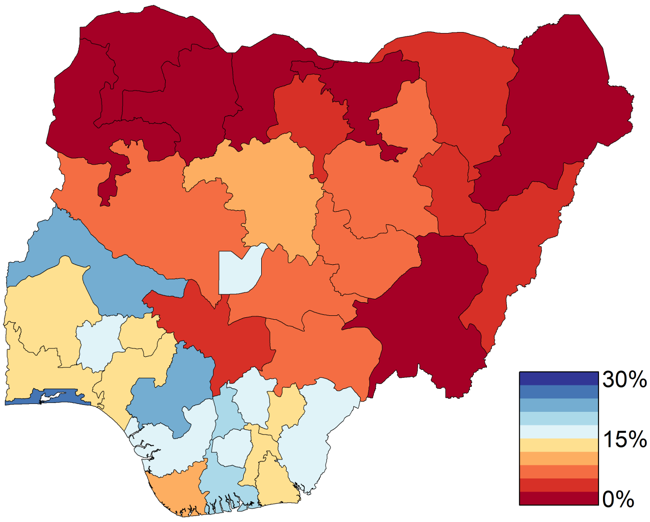

(b) Age 15-24 years and parity 1+.

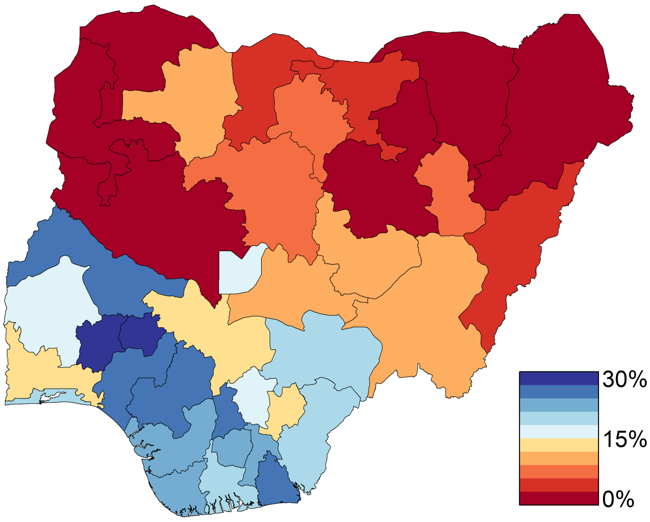

(c) Age 25-49 years and parity 0.

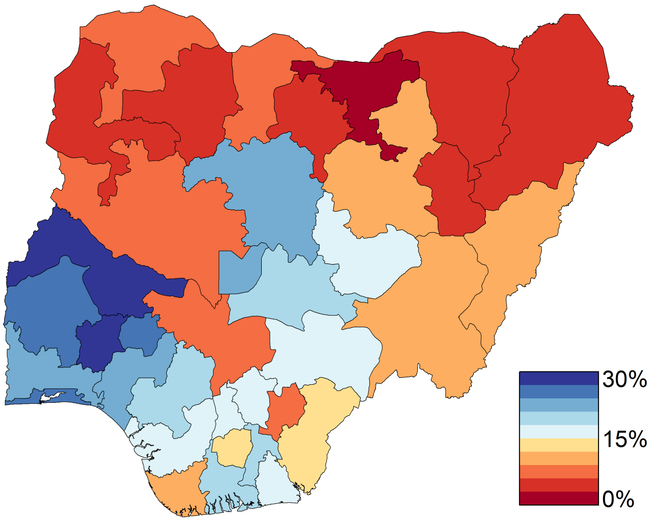

(d) Age 25-49 years and parity 1+.

Figure 8: Smoothed estimates of mCPR by age and parity group for 2017.

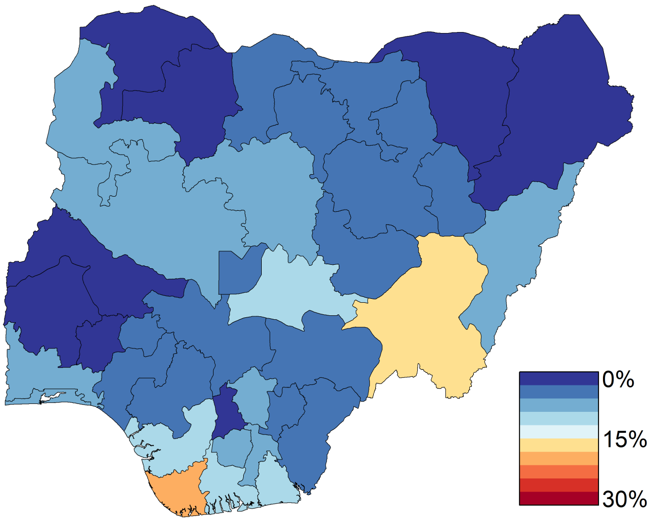

(a) Age 15-24 years and parity 0.

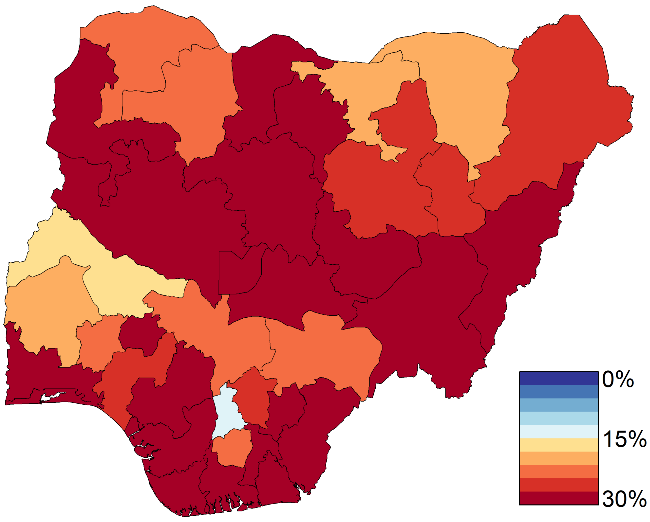

(b) Age 15-24 years and parity 1+.

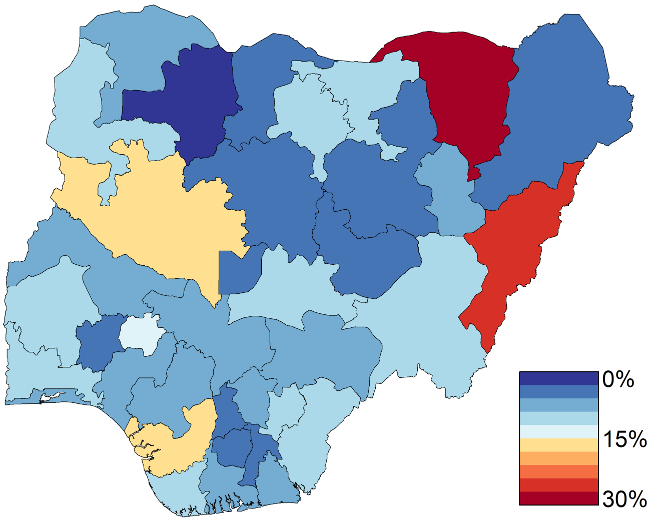

(c) Age 25-49 years and parity 0.

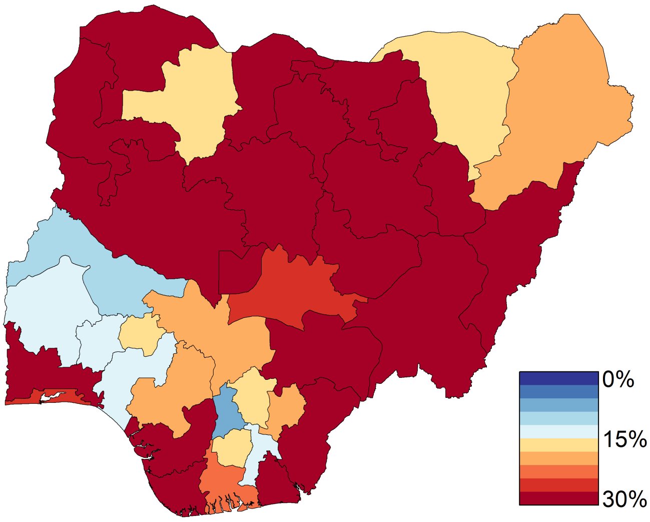

(d) Age 25-49 years and parity 1+.

Figure 9: Smoothed estimates of unmet need by age and parity group for 2017.

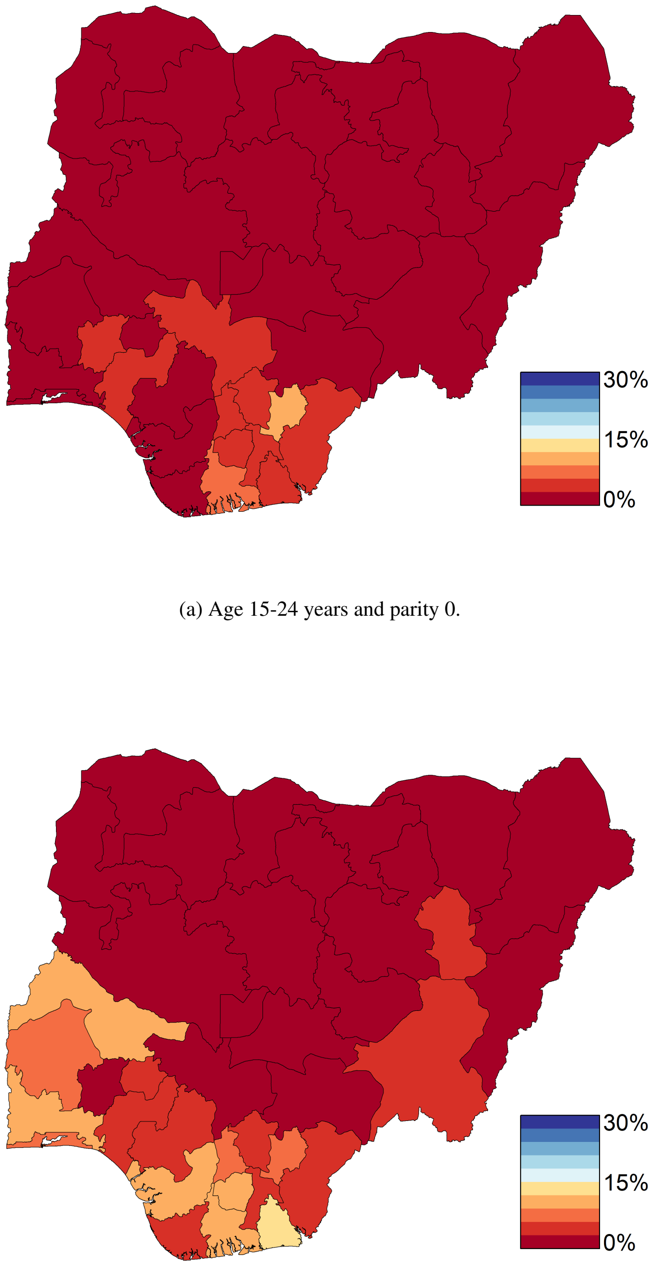

(a) Age 15-24 years and parity 0.

(b) Age 15-24 years and parity 1+.

(c) Age 25-49 years and parity 0.

(d) Age 25-49 years and parity 1+.

Figure 10: Smoothed estimates of traditional CPR by age and parity group for 2017.

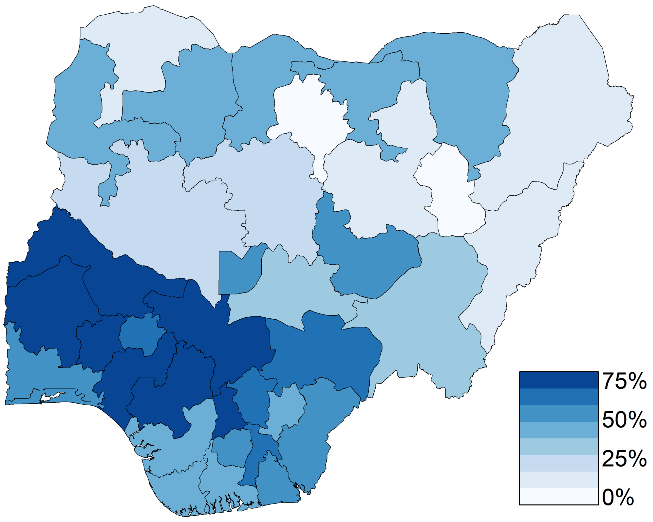

(a) Age 15-24 years and parity 0.

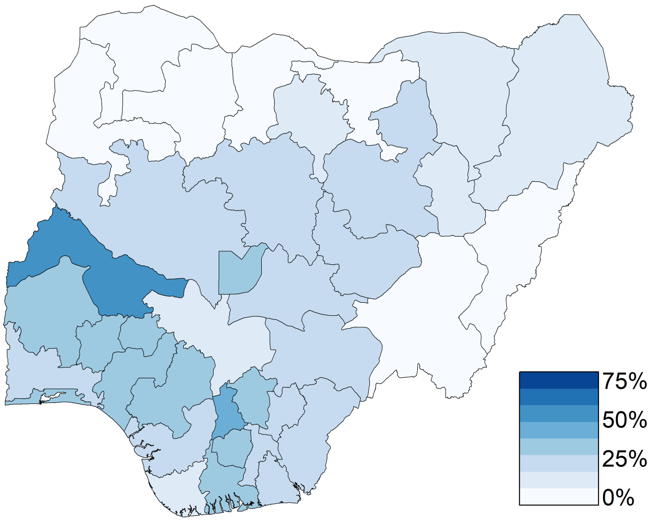

(b) Age 15-24 years and parity 1+.

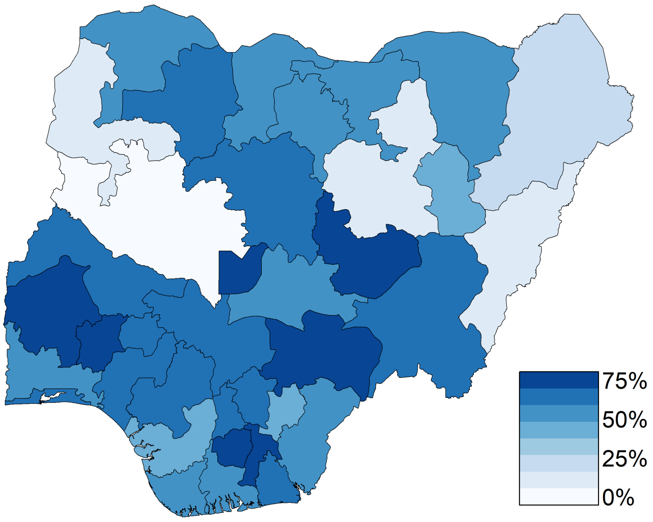

(c) Age 25-49 years and parity 0.

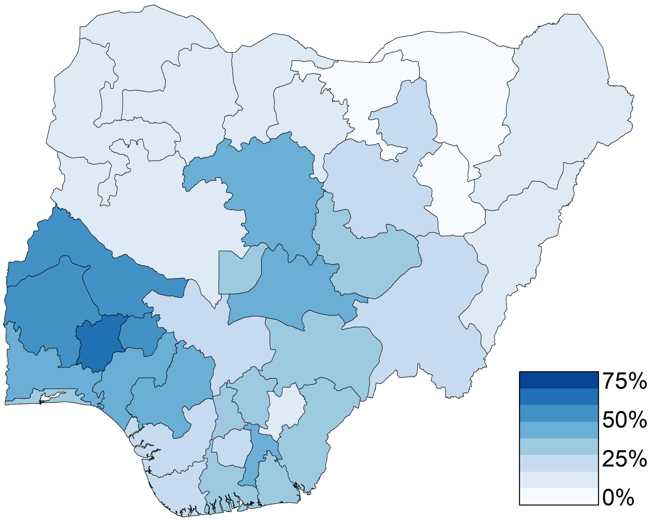

(d) Age 25-49 years and parity 1+.

Figure 11: Smoothed estimates of demand satisfied by age and parity group for 2017.

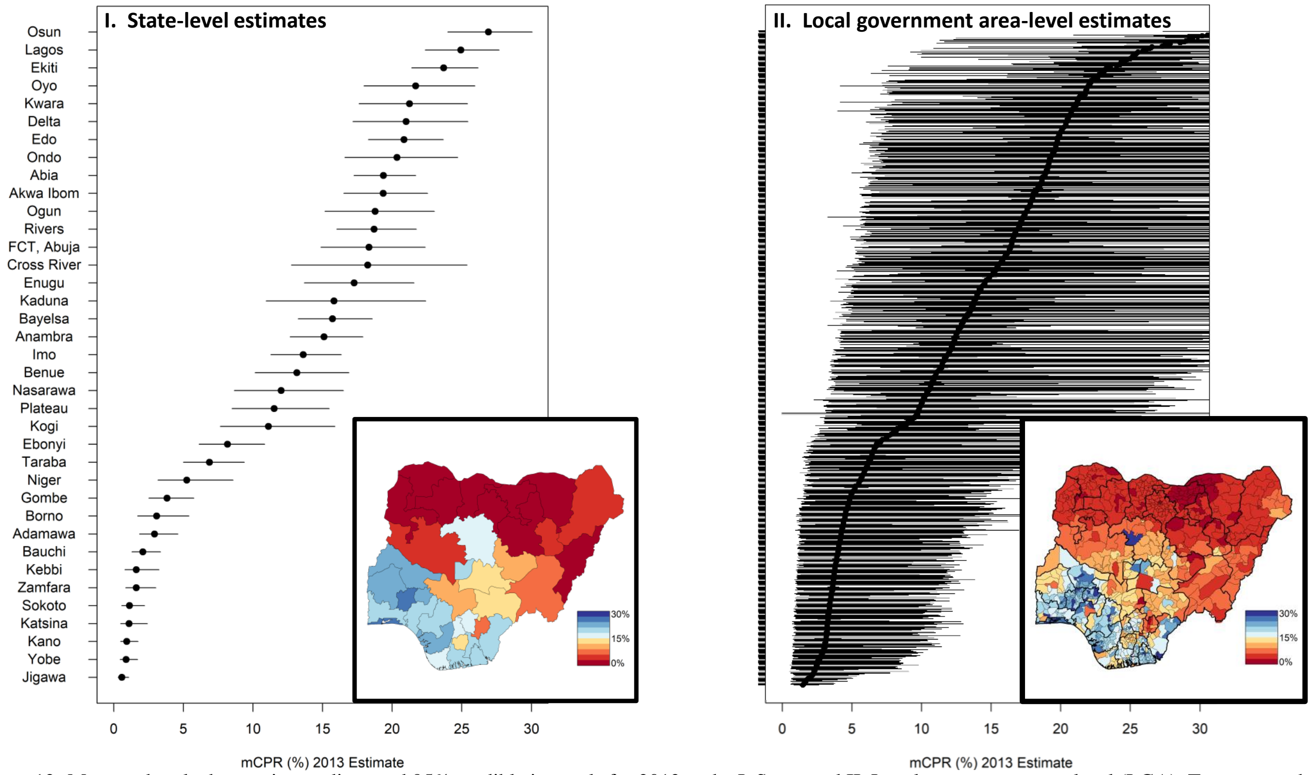

Figure 12: Maps and ranked posterior medians and 95% credible intervals for 2013 at the I. State- and II. Local government area-level (LGA). To generate the map of Nigeria at the LGA level, we utilized the publicly available shapefiles at [30].
